# Childhood-onset schizophrenia: what do we really know?

**DOI:** 10.1080/21642850.2014.927738

**Published:** 2014-07-15

**Authors:** Jennifer Bartlett

**Affiliations:** ^a^ Department of Educational Psychology, University of Alberta, Edmonton, AB, CanadaT6G 0X6

**Keywords:** mental health and disorder, stigma or discrimination, literature review and meta-analysis, children and adolescents

## Abstract

Childhood-onset schizophrenia (COS) is a rare, chronic mental illness that is diagnosed in children prior to the age of 13. COS is a controversial diagnosis among clinicians and can be very difficult to diagnose for a number of reasons. Schizophrenia is a psychotic disorder characterized by hallucinations, delusions, flat affect, limited motivation and anhedonia. The psychotic nature of this disorder is quite disruptive to the child's emotional regulation, behavioural control and can reduce the child's ability to perform daily tasks that are crucial to adaptive functioning. Prior to the onset of schizophrenia, children often develop premorbid abnormalities, which are disturbances to a child's functioning that may serve as warning signs. These disturbances can manifest in a variety of behavioural ways and may include introversion, depression, aggression, suicidal ideation and manic-like behaviours. This article will review the clinical presentation of schizophrenia in children and examine the existing knowledge around aetiology, treatment approaches, assessment techniques and differential diagnostic considerations. Gaps in the literature are identified and directions for future research are discussed.

## Introduction

Schizophrenia is a chronic mental illness characterized by two categories of symptoms: positive and negative. Positive symptoms include hallucinations, delusions, disorganized thinking and speech patterns and abnormal motor behaviour, which may include bizarre movements or catatonia (American Psychiatric Association, 2013; Coghill, Bonnar, Duke, Graham, & Seth, 2009). Negative symptoms include blunt or flat affect, lack of motivation, absence or diminished speech patterns, diminished interest in social interaction and anhedonia (American Psychiatric Association, 2013; Coghill et al., 2009). Schizophrenia most commonly emerges during early adulthood between the ages of 16 and 30, but it can also be diagnosed during childhood (The National Institute of Mental Health, 2009). A diagnosis of childhood-onset schizophrenia (COS) is given when the onset of the illness occurs prior to age 13 (Remschmidt et al., 2006; Sharma & Harvey, 2006). COS is a very rare illness and as such is poorly understood. This lack of understanding makes it difficult to accurately diagnose and, as a result, children with schizophrenia are often misdiagnosed. It is important that clinicians have an in-depth understanding of the manifestation and prognosis of COS in order to better recognize and treat it (Taylor, 1998). In addition to clinicians, it is also important that individuals and families struggling with schizophrenia are educated so that they can be better prepared to cope with it (Mayo Foundation for Medical Education and Research, 2013). COS has an estimated prevalence rate of approximately 1/10,000, and tends to occur more often in males than in females (Coghill et al., 2009; Sood & Kattimani, 2008). Although the presentation can be quite different in children, COS is diagnosed according to the same criteria for adult-onset schizophrenia with the exception of Criteria B. Criteria B states that the afflicted individual's level of functioning must be diminished. However, as this is difficult to assess in children, they instead must fail to meet the expected level of functioning for a child according to their age (American Psychiatric Association, 2013).

COS is very difficult to accurately diagnose, and as such many clinicians are reluctant to do so. One of the key difficulties in making this diagnosis is distinguishing between true hallucinations and delusions and a child's imaginative play (Coghill et al., 2009; Taylor, 1998). For example, many children have imaginary friends which may be mistaken for psychosis. Similarly, children with poor or underdeveloped language skills may mimic the disorganized thought and speech patterns observed in schizophrenia (Coghill et al., 2009). Children may be unable to reliably describe their experiences and symptoms due to a restricted vocabulary or a limited understanding of their internal experiences. As a result, healthcare professionals may be unable to collect the information needed to make a diagnosis (Taylor, 1998).

In cases of COS, there are often disturbances in the child's psychosocial functioning prior to the onset of the illness, which are referred to as premorbid abnormalities. Premorbid abnormalities can include a range of behaviours such as shyness, introversion, loneliness, depression, aggression, suicidality, theft and manic-like or bizarre behaviour (Eggers, Bunk, & Krause, 2000). One of the most commonly reported initial presenting issues in children is that they are struggling in school, which may be a direct result of the behavioural difficulties that arise in COS (Eggers et al., 2000; Schaeffer & Ross, 2002). Problematic behaviours are typically noted upon entering school at age 5 or 6, although families often report that the disruptive behaviours began prior to schooling (Schaeffer & Ross, 2002). As adult-onset schizophrenia develops between the ages of 16 and 30, premorbid abnormalities are not observed in these patients. However, some patients do experience prodromal symptoms prior to the active phase of schizophrenia, which are simply a mild form of hallucinations or delusions. Both children and adults with schizophrenia can experience the range of positive and negative symptoms, although children's delusions and hallucinations may be less complex than those of adults (American Psychiatric Association, 2013).

The diagnosis of COS is thought to be on a clinical continuum with adult-onset schizophrenia and appears to be relatively stable across time, continuing into adulthood (Hollis, 2000; Sharma & Harvey, 2006). Remschmidt et al. (2006) conducted a long-term study following 16 patients who were diagnosed with COS over the course of 42 years. At the time of follow-up, patients were assessed using a Global Assessment Scale revealing that the majority of patients had a poor outcome overall. The majority of patients displayed severe to moderate depressive symptoms, failed to graduate from secondary school or secure employment, and had a higher suicide rate than the general population. The majority of the patients displayed negative symptoms and a minority displayed positive symptoms. Overall, the long-term diagnostic stability of this set of patients was 91%, with only 7 of 16 patients having received an alternate diagnosis.

The prognosis of COS is generally quite poor (Eggers et al., 2000). Eggers et al. (2000) conducted a long-term study to examine the outcomes of 11 patients with COS. At the time of follow-up, approximately 41 years later, some clients were in a state of partial remission while others were not. The majority of patients experienced multiple schizophrenic episodes lasting only a short time, but two patients in particular had schizophrenic episodes that were of extended duration. These episodes included two catatonic episodes lasting 30 and 40 years, a paranoid episode of 30 years and a disorganized episode lasting 42 years. The majority of patients had poor social adjustment, and evidence suggests that patients who experienced catatonic episodes had the poorest social adjustment overall.

## Aetiology

The underlying causes in the development of COS are varied and poorly understood. Furthermore, while there is research on the factors that contribute to the development of schizophrenia in general, very few studies have focused their investigation on COS. However, as COS is thought to be continuous with adult-onset schizophrenia and is a strong predictor of a continuation of the illness into adulthood, the mechanisms underlying the two are thought to be the same (Sharma & Harvey, 2006). As outlined in Table 1, the most common aetiological risk factors include familial factors, obstetric and pre-natal complications, genetics and neurodevelopment.

**Table 1.** Risk factors.

Risk factors
Chromosomal deletions on chromosomes 1, 8, 15 and 22
Too few neural connections
Too many neural connections
Altered functionality of neurotransmitters: dopamine, serotonin, glutamine and GABA
Paternal parents aged 30 and older at the time of conception
Family history of schizophrenia spectrum disorders and/or personality disorders

Note: A summary of the aetiological risk factors for developing COS.

### Familial factors

Very little research has investigated the impact of familial factors on the development and maintenance of COS. Evidence suggests that adverse experiences and negative familial interactions can contribute to the development of schizophrenic symptoms (Gallagher & Jones, 2013). More specifically, childhood neglect, such as being ignored or rejected, is associated with the development of negative symptoms. On the other hand, childhood mistreatment, such as physical or sexual abuse, is associated with the development of positive symptoms (Gallagher & Jones, 2013).

### Obstetric and pre-natal complications

There is evidence to suggest that pre-natal infection can increase the likelihood of offspring developing schizophrenia in both childhood and adulthood (Brown et al., 2004; Coghill et al., 2009; Sharma & Harvey, 2006). Pre-natal exposure to the influenza virus during the first trimester of pregnancy appears to increase the risk of developing schizophrenia, making the offspring seven times more likely to develop this illness. However, pre-natal exposure during the second and third trimesters does not appear to increase the risk of developing schizophrenia (Brown et al., 2004). In addition to the influenza virus, pre-natal exposure to the Rubella virus, respiratory infection, analgesics and malnutrition have also been shown to increase the risk of developing COS (Clarke, Harley, & Cannon, 2006; Coghill et al., 2009; Sharma & Harvey, 2006). Lastly, obstetric complications during childbirth have also been identified as a risk factor for the development of COS. The experience of hypoxia in offspring during childbirth, a phenomenon where the body is deprived of oxygen for some period of time, increases the likelihood that offspring will develop COS (Coghill et al., 2009; Sharma & Harvey, 2006).

### Genetics

Evidence suggests there is a significant heritable component to COS. There are multiple genes and genetic mutations that have been hypothesized as being integral to COS and its hereditary nature (Coghill et al., 2009).

Although it is not specific to COS, the age of the paternal parent is one risk factor that has been identified in the development of schizophrenia. More specifically, a paternal parent with a more advanced age over the age of 30 at the time of conception appears to be associated with offspring developing schizophrenia. Furthermore, the older the paternal parent is, the more likely the offspring are to develop schizophrenia (Sipos et al., 2004; Zammit et al., 2003). It is hypothesized that this association exists due to a cell mutation that occurs as paternal age increases, although this mechanism is not well understood (Zammit et al., 2003).

There is also evidence to suggest that a family history of mental illness may increase the likelihood of offspring developing COS. In one sample of COS patients, approximately 80% of those patients had a family history of psychiatric disorders. More specifically, this association appears to exist only if the family has a history of either schizophrenia spectrum disorders or personality disorders (Margari et al., 2011). Interestingly, parents of children with schizophrenia are 10 times as likely to develop schizophrenia themselves (Coghill et al., 2009).

It is clear that there is a genetic contribution to the development of COS, but the nature of this contribution is poorly understood. Several studies have attempted to identify specific genes that may be associated with or serve as risk factors for the development of COS. One study identified up to 94 genes that were thought to be involved in the development of schizophrenia, which act through different biological pathways. The majority of these genes were related to the functionality of several neurotransmitters, or chemical signals within the brain, including dopamine, serotonin, glutamate and gamma-aminobutyric acid (GABA) (Greenwood et al., 2011). The genes associated with neurotransmitter signalling are thought to mediate susceptibility to schizophrenia, although it is unclear how (Greenwood et al., 2011). Lastly, there are certain chromosomal deletions that are thought to be associated with the development of COS, including deletions on chromosomes 1, 8, 15 and 22 (Coghill et al., 2009; The International Schizophrenia Consortium, 2008).

### Neurodevelopmental model

The neurodevelopmental model of COS outlines the various structural, pathological and functional nuances of the brain that are associated with this illness, as well as the resulting cognitive implications.

During the process of neurodevelopment, the brain develops an excess of neural connections. As brain development progresses into adolescence, the brain changes by eliminating unnecessary and unused connections (Coghill et al., 2009; Sharma & Harvey, 2006). Post-mortem studies suggest that, among patients with schizophrenia, there are an abnormal number of neural connections. It is hypothesized that both hyper-aggressive and hypo-aggressive removal of neural connectivity can lead to the development of psychosis (Sharma & Harvey, 2006). There may be a greater reduction in neural connections than is considered normal, resulting in less connectivity and brain activity. Conversely, there may be too few neural connections eliminated, indicating excessive connectivity and brain activity (Coghill et al., 2009; Sharma & Harvey, 2006).

In COS, evidence suggests that the brain's ventricles can become enlarged which has been associated with a form of COS that is more difficult to treat and results in a much poorer outcome (Coghill et al., 2009; Sharma & Harvey, 2006). There is also a relationship between enlarged ventricles and the presence of persistent positive and negative symptoms. The causal direction of this relationship remains to be unclear; the persistence of these symptoms may directly result in changes to the brain's structure, or the brain's structure may give rise to persistent psychotic symptoms (Sharma & Harvey, 2006).

Similarly, it has been hypothesized that loss of grey brain matter as is typical in COS may in fact trigger the onset of this illness during childhood (Sharma & Harvey, 2006). A decrease in total cerebral volume occurs as a result of the gradual decline of grey matter, which has also been shown to be associated with poor premorbid functioning in children, prior to the onset of schizophrenia (Coghill et al., 2009; Sharma & Harvey, 2006). The presence of grey matter, particularly in large quantities, is associated with a higher premorbid IQ among children (Sharma & Harvey, 2006) which suggests that lower levels of intelligence in COS may be the direct result of the loss of grey matter characteristic of this illness.

Lastly, evidence suggests that the negative symptoms of schizophrenia are linked to a dysfunction of the brain's frontal lobe, indicating a deficit in executive functioning, such as memory, reasoning, problem solving and planning (Coghill et al., 2009; Sharma & Harvey, 2006). Positive symptoms, on the other hand, appear to be linked to a dysfunction within the temporal lobe of the brain, which may indicate deficits in memory, executive functioning, verbal expression and abstract thinking (Sharma & Harvey, 2006).

While it is clear that there are distinct abnormalities in the structure of the brain with COS, the factors that may trigger this abnormal development remain a mystery.

## Differential diagnosis

The presence of overlapping symptomology and co-morbid disorders and the early age at which children experience psychotic-like symptoms can make it very difficult to accurately diagnose COS. One study showed that among a sample of 17 patients with COS, there were a total of 43 alternative diagnoses given prior to diagnosing schizophrenia (Schaeffer & Ross, 2002). Similarly, a five-year-old boy who experienced auditory hallucinations received a multitude of inaccurate diagnoses before being formally diagnosed with schizophrenia. The alternate diagnoses included pervasive developmental disorder, attention deficit hyperactivity disorder (ADHD), bipolar disorder (BD), major depressive disorder and schizoaffective disorder (Schaeffer & Ross, 2002).

High rates of comorbidity have been found among patients with COS, particularly with ADHD and affective disorders, which makes differential diagnosis a crucial factor for consideration with this population (Ross, Heinlein, & Tregellas, 2006). There are a number of disorders that must be considered in the differential diagnosis of COS, including major depressive disorder, schizoaffective disorder, schizophreniform disorder, brief psychotic disorder, delusional disorder and schizotypal personality disorder. According to the literature, the three most common disorders that overlap with and are difficult to distinguish from COS are BD, autism spectrum disorder (ASD) and ADHD (Table 2) (Dossetor, 2007; Ross et al., 2006; Schaeffer & Ross, 2002; Tahiroğlu, Çelik, & Avci, 2009).

**Table 2.** Differential diagnosis.

Disorder	Commonalities	Distinguishing features
BD	• Can present with psychotic symptoms (delusions, hallucinations, disorganized behaviour, catatonia and paranoia)	• Mood congruent delusions
		• Delusions/hallucinations occur exclusively during depression or mania
ASD	• Disorganized speech	• No hallucinations or delusions
	• Flat affect	• Lack of atypical beliefs
	• Social deficits	
	• Repetitive and bizarre movements and behaviours	
ADHD	• Poor attention	• Absence of psychotic episode
	• Disorganized	

Note: A summary of the similarities and differences between schizophrenia and BD, ASD and ADHD to aid in making a differential diagnosis.

BD in children can present with psychotic symptoms, thus sharing clinical features with COS (Pavuluri, Herbener, & Sweeney, 2004; Tahiroğlu et al., 2009). The most common psychotic symptoms present in children with BD are mood congruent and grandiose delusions (Pavuluri et al., 2004). This is an important distinction in the differential diagnosis of COS because, while the psychotic symptoms in BD appear to be consistent with the patient's affect, they occur in COS independent of affect (Pavuluri et al., 2004). A distinguishing feature between COS and BD is that, if delusions or hallucinations occur exclusively during periods of either depression or mania, then the recommended diagnosis is BD with psychotic features (American Psychiatric Association, 2013). Both COS and BD can present with changes in mood; where schizophrenia may present with elements of depression, BD can include both low and elated moods and irritability. Clinicians need to be aware of this overlap and carefully consider the presentation of affect before diagnosing COS. Additionally, BD may also present in children with audio, visual and tactile hallucinations, disorganized behaviour, catatonia and paranoia (American Psychiatric Association, 2013; Tahiroğlu et al., 2009).

Examining the mental state of a child can be difficult, but this becomes further complicated in the presence of a developmental delay. COS is sometimes misdiagnosed in cases of ASD due to the similarities in symptom presentation (Dossetor, 2007). The symptoms of ASD, such as disorganized speech and flat affect, can mimic the negative symptoms observed in COS (Dossetor, 2007). The social deficits seen in ASD may also be confused with the social impairment and atypical beliefs that children with schizophrenia often display (American Psychiatric Association, 2013). Children with autism often demonstrate stereotyped movements, thoughts, behaviours and interests. The degree of repetitiveness can appear bizarre, and it becomes difficult to distinguish whether or not they are the result of psychosis (Dossetor, 2007). A child with ASD can also be comorbidly diagnosed with COS, but only if there are prominent hallucinations or delusions present for at least one month (American Psychiatric Association, 2013).

Although there appears to be very little research on the differential diagnosis of ADHD from COS, it has been identified as a common misdiagnosis in cases of COS and is therefore an important disorder to distinguish from schizophrenia (Coghill et al., 2009; Ross et al., 2006; Schaeffer & Ross, 2002; Sharma & Harvey, 2006). It has been noted that among the many presenting symptoms of COS, attentional difficulties are commonly seen in children (Coghill et al., 2009; Schaeffer & Ross, 2002; Sharma & Harvey, 2006). Research suggests that the degree and frequency of impaired attention observed in COS is equal to the inattention found in ADHD-combined type, which includes aspects of both inattentive ADHD and hyperactive-impulsive ADHD (Egeland, 2010). The diagnosis of ADHD should not be made if inattention or hyperactivity occur exclusively alongside a psychotic episode or disorder (American Psychiatric Association, 2013).

## Assessment

The assessment of COS can be very complex and requires both formal testing and observation. Unfortunately, there is no one test or procedure that can determine the presence of COS, which is one of the many reasons why schizophrenia is difficult to diagnose in children.

There are tools that were developed to assess the presence and severity of COS, such as the Schedule for Affective Disorders and Schizophrenia, the Positive and Negative Symptom Scale and the Premorbid Adjustment Scale. The Schedule for Affective Disorders and Schizophrenia is a semi-structured diagnostic interview and has relatively good convergent and divergent validity (Coghill et al., 2009; Lauth et al., 2010). The Positive and Negative Symptom Scale is a robust tool used in the assessment of schizophrenia and related disorders (Coghill et al., 2009; Linden, Scheel, & Rettig, 2007). This scale is useful in evaluating the severity of the illness and is relatively easy to use (Linden et al., 2007). Lastly, the Premorbid Adjustment Scale allows for the assessment of premorbid functioning, as research indicates that certain premorbid symptoms are associated with the development of COS, and has good predictive and concurrent validity (Brill, Reichenberg, Weiser, & Rabinowitz, 2008; Eggers et al., 2000). The Premorbid Adjustment Scale can be used to assess for schizophrenia during childhood, adolescence and adulthood (Shapiro et al., 2009).

Neuropsychological assessments are also used in the identification of COS, particularly positron emission tomography (PET), single photon emission computed tomography (SPECT) and magnetic resonance imaging (MRI) (Coghill et al., 2009; Malhotra, Gupta, Bhattacharya, & Kapoor, 2006). Both PET and SPECT work by injecting a tracer into the patient that will allow for a visual examination of the physiological functioning of the brain by tracing blood flow (Malhotra et al., 2006). MRI is a neuroimaging technique that allows for the examination of brain structure, rather than function, and can aid in the identification of structural abnormalities (Coghill et al., 2009). If these tests yield functional and structural results consistent with the brain abnormalities typically found in COS, then this illness may be present and further testing should be done.

In conjunction with psychometric and neuropsychological testing, a psychiatric assessment should also be completed. Psychiatric assessments are more subjective and include a thorough examination of the various areas of development and functioning in a child's life. The areas that must be assessed include the child's developmental history such as their achievement of developmental milestones, family history of mental illness, a close examination of the child's mental state with particular emphasis on the presence or absence of psychotic symptoms, risk posed to the self or others, family functioning and medical history (Coghill et al., 2009). As COS is challenging to diagnose, clinicians may need to enlist a variety of assessment tools before issuing a formal diagnosis.

## Treatment

Treatment of COS requires an interdisciplinary team of healthcare professionals, which may include psychiatrists, psychologists, paediatricians, social workers and psychiatric nurses (Mayo Foundation for Medical Education and Research, 2013). The primary form of treatment for schizophrenia, including COS, is antipsychotic medication (Mayo Foundation for Medical Education and Research, 2013). Although very few studies have examined the efficacy and safety of antipsychotic use in children, they continue to be the first line of treatment for schizophrenia with this population (Mayo Foundation for Medical Education and Research, 2013). As outlined in Table 3, there are two categories of antipsychotic medications, typical and atypical, which differ in efficacy and side effects (Mayo Foundation for Medical Education and Research, 2013). Typical antipsychotics, such as haloperidol and loxapine, also known as first generation antipsychotics, were the first antipsychotics to be developed (Armenteros & Davies, 2006). Typical antipsychotics are generally known as having the most adverse side effects, the most severe of which are the motor and movement disorders that can cause involuntary movements of the face, tongue, limbs and hands (Mayo Foundation for Medical Education and Research, 2013). Additional side effects may include weight gain, restlessness, anxiety, irritability and drowsiness (Sikich et al., 2008). Due to the severity of these side effects, typical antipsychotics are one of the last forms of treatment used with childhood schizophrenia (Mayo Foundation for Medical Education and Research, 2013).

**Table 3.** Forms of treatment.

Atypical antipsychotics	Typical antipsychotics	Alternatives
Clozapine	Haloperidol	Individual therapy
Risperidone	Loxapine	Family therapy
Olanzapine	Molindone	Skill building and psychoeducation

Note: A summary of the types and forms of treatment available for COS.

The severity of typical antipsychotics are what led to the development of second generation, or atypical, antipsychotics. Atypical antipsychotics are usually the first medications used in the treatment of COS as they tend to have fewer side effects than their counterparts (Mayo Foundation for Medical Education and Research, 2013). The side effects of atypical antipsychotics include weight gain, diabetes, high cholesterol, seizures and, more rarely, movement disorders (Armenteros & Davies, 2006; Mayo Foundation for Medical Education and Research, 2013; Sikich et al., 2008).

Clozapine, Risperidone and Olanzapine are among the most commonly prescribed and researched atypical antipsychotics for the treatment of schizophrenia. Clozapine, in particular, is argued to be the most effective atypical antipsychotic medication for treating psychosis, and has been shown to be effective in treatment-resistant schizophrenia (Kumra et al., 2008; Shaw et al., 2006). However, Clozapine can have particularly dangerous side effects when used to treat children with schizophrenia, the most severe of which is neutropenia. Neutropenia is when a significant drop in white blood cell count occurs. Among a sample of 87 children and adolescents being treated with Clozapine, approximately half of them displayed mild to moderate neutropenia, which is much higher than the risk of neutropenia among adults. There appears to be certain risk factors that can increase the likelihood of developing this complication, including younger age, being male and being an African-American (Maher et al., 2013). There continues to be disagreement around the efficacy of typical versus atypical antipsychotic medication in the treatment of schizophrenia. Where some studies suggest that typical antipsychotics are more efficacious than atypical, others indicate there are no significant differences in their treatment of COS.

One study conducted a meta-analysis of 15 studies examining the effectiveness of typical and atypical antipsychotics. This meta-analysis revealed that children responded to atypical antipsychotics 55% of the time, in comparison to a response rate of 72% for typical antipsychotics. This study suggests that typical antipsychotics have greater efficacy in treating childhood schizophrenia than atypical antipsychotics (Armenteros & Davies, 2006). Another study compared the efficacy of two atypical antipsychotics with one typical antipsychotic in the treatment of childhood schizophrenia. Children appeared to respond most favourably to the typical antipsychotic with a response rate of 50%. However, this response rate was not significantly different from the response rates to the two atypical antipsychotics which were 34% and 46%. This suggests that both typical and atypical antipsychotics are equally effective in treating childhood schizophrenia (Sikich et al., 2008). These findings are consistent with subsequent research into the efficacy of antipsychotic medications, which indicate that both typical and atypical antipsychotics show similar reductions in the symptoms of schizophrenia with no one class of drug demonstrating greater or lesser efficacy (Findling et al., 2010).

It is clear that there is a lack of information and a need for further study on the efficacy of typical and atypical antipsychotic medication in the treatment of COS. The majority of these medications have not been approved by the Food and Drug Association for use on children, with the exception of Trifluoperazine, which has been approved for the treatment of schizophrenia in children between the ages of 6 and 12 years old, and Thioridazine (Maloney, Yakutis, & Frazier, 2012). However, despite this lack of knowledge, antipsychotic medication continues to be prescribed to children with schizophrenia. This is an important body of work that needs to be studied in order to educate health practitioners on the best practices in treating COS, and to enhance our understanding of the impact these medications, which were designed for adult use, can have on children.

Although their efficacy has not been investigated to date, there are also therapeutic interventions that can be used in treating schizophrenia (Table 3) including individual therapy, family therapy and social skills training (Mayo Foundation for Medical Education and Research, 2013).

Individualized therapy with children can reduce their symptoms by helping them learn how to cope with the various stressors and challenges of living with schizophrenia. Therapy can help children improve their academic success, resolve difficulties at school and improve their ability to maintain relationships with peers (Mayo Foundation for Medical Education and Research, 2013).

Family therapy focuses on providing not only the child but the family unit with support and education in coping with a child's illness. Family members can then in turn support their children in working with their illness. Additionally, families can learn to improve their communication skills, work through family conflict and cope with family stress as related to the child's mental health (Mayo Foundation for Medical Education and Research, 2013). When using family-based interventions to improve schizophrenia, the families’ emotions are the catalysts for change (Kuipers, 2006). Families in which one or more members are suffering from schizophrenia are often characterized by high levels of anxiety and criticism. The goal of family therapy is to educate families about the illness, help them reduce their stress, develop coping techniques and improve their ability to problem solve. Working with the family unit rather than the individual child is beneficial because it can help the family develop a more positive style of interaction. Family members often fill the role of the caregiver and, as a result, may themselves suffer from depression, anxiety, feel burdened and become critical or negative towards the child with schizophrenia. Family therapy is intended to help family members improve their own mental health, develop skills to effectively cope with their own anxiety as well as the mentally ill family member and adopt a less critical attitude. By improving the family dynamic, the goal is that the individual with schizophrenia will in turn have lower anxiety and depression, thus decreasing the likelihood of a relapse (Kuipers, 2006). Family intervention for individuals with schizophrenia has been shown to reduce patient relapse rates, both during and following treatment, and appears to reduce the likelihood that patients will require hospitalization during the course treatment. It has shown to be particularly effective for schizophrenic patients who recently experienced a relapse or who present with persistent symptoms (NICE, 2003). It is unclear as to why family therapy may be helpful in the treatment of Schizophrenia, just as it is unclear as to how empirically efficacious it is in comparison to other treatment options. It has been hypothesized that family interventions work primarily due to the shift in family affect, which in turn can impact the individual with schizophrenia. A patient with a family that primarily expresses negative affect is more likely to suffer from higher levels of anxiety and depression, which may trigger a psychotic episode (Kuipers, 2006).

Lastly, teaching children social and academic skills is an important component to managing schizophrenia. Children with schizophrenia often struggle with interpersonal relationships and academic success, as well as everyday tasks including bathing and dressing themselves. The primary goal of this training is to provide children with skills that can improve their daily functioning (Mayo Foundation for Medical Education and Research, 2013).

## Controversy

Diagnosing schizophrenia in childhood is a topic of controversy as it is a poorly understood illness and is incredibly rare (Sood & Kattimani, 2008). Given the difficulty in accurately diagnosing schizophrenia in children, the question becomes: Should clinicians be diagnosing COS at all?

The literature tells us that it is not uncommon for COS to be misdiagnosed when in fact another diagnosis may be more appropriate, such as BD or ASD (Dossetor, 2007; Kempf, Hussain, & Potash, 2005; Pavuluri et al., 2004; Tahiroğlu et al., 2009). When children are misdiagnosed with schizophrenia, they are likely to be exposed to a range of unnecessary and ineffective treatments, the most harmful of which are pharmacological. A child diagnosed with schizophrenia will be treated as such, most likely through the use of antipsychotic medication. However, if this diagnosis is inaccurate then antipsychotics will not provide them with the help they need, and may in fact cause damage as they are known to have severe side effects including weight gain and movement disorders (Armenteros & Davies, 2006; Sikich et al., 2008). Therefore, if a diagnosis is so difficult to confirm and the method of treatment is so severe, then it is arguably better to withhold such a diagnosis and look for alternative ways of helping the child.

Indeed many clinicians are reluctant to diagnose children with schizophrenia due to the complex nature of the illness. However, children with schizophrenia who are wrongly given alternate diagnoses due to a clinician's reluctance to diagnose COS will also suffer the effects of mis-medication. Similarly, if a clinician withholds a diagnosis of COS when a child in fact fits the diagnostic criteria, then they may be denying both the child and the family the treatment and support that they need (Coghill et al., 2009). While it is important that clinicians use extreme caution in assessing and diagnosing COS, it is arguably unethical to not appropriately diagnose a child who meets the diagnostic criteria for an illness due to a personal bias.

While there is no right or wrong answer to this controversial topic, it is clear that clinicians must demonstrate due diligence and be vigilant when considering the diagnosis of COS. The diagnosis of COS should be given when appropriate, but may require the clinician to carefully assess the client over an extended period of time before coming to that conclusion.

## Concluding remarks

Antipsychotics are commonly used to treat children with COS despite the fact that they can have very severe and debilitating side effects (Armenteros & Davies, 2006; Sikich et al., 2008). Although they appear to be an effective form of treatment, research on COS has not examined how antipsychotic medications may affect the continuing development of children throughout life. Given that it is difficult to accurately diagnose COS and that the effects of antipsychotic medication on childhood development are unclear, it may be beneficial to examine safer alternative methods of treating psychosis beyond pharmacological intervention. Future research should aim to bridge these gaps by focusing on the neurodevelopmental and behavioural impact that antipsychotic medications can have on a developing child.

There continues to be significant gaps in the literature on the underlying causes of COS, and it is clear that further research needs to be done on this topic. While genetic and neurodevelopmental contributions have been examined, very little research has been done on the contribution of family dynamics and the home environment to the development, maintenance, severity and treatment of COS. Research indicates that family therapy can be used in the treatment of COS, and it has been hypothesized that this treatment may be effective by changing the family's affect, interactions, anxiety and criticisms (Kuipers, 2006). However, research has not yet adequately addressed the efficacy of this treatment. One study showed that among a sample of families who sought non-pharmacological treatment for COS, a minority of 33% found this treatment to be effective (Schaeffer & Ross, 2002). Although the majority of families did not find therapy helpful, approximately one-third did benefit from it, suggesting that this may be an avenue for future research and treatment. Additionally, if family therapy is in fact effective in improving COS, then this suggests that there are certain aspects of the familial environment that can promote or exacerbate this condition.

There appears to be many shared features in the presentation of COS and bipolar disorder. Schizophrenia can present with affective features just as BD can present with psychotic features (Dossetor, 2007; Pavuluri et al., 2004; Ross et al., 2006). Evidence suggests that these disorders have overlapping genetic markers and neurotransmitter dysfunction. Additionally, research is now demonstrating the successful use of atypical antipsychotics in the treatment of BD (Gentile, 2011; Möller, 2003). The degree to which these two disorders overlap suggests that they may not be entirely distinct from one another, but may in fact be on a continuum where schizophrenia and related disorders are at one end, and affective disorders are at the other. There is in fact emerging evidence to support this claim, although further research needs to be done (Keshavan et al., 2011). The observed hereditary overlap also raises the question as to whether schizophrenia and BD share a similar aetiology, a hypothesis that may warrant future study.

While COS remains somewhat of a mystery, a foundational knowledge of aetiology, treatment and challenges in diagnosis has been established. However, there continues to be gaps in our understanding of the mechanisms through which COS can arise as well as best practices in distinguishing and diagnosing COS from other childhood disorders. Future research should address these gaps, as well as the implication of the family environment in the development and maintenance of COS.
